# Autograft vs. Xenograft Duraplasty Using the Onlay Technique in Pediatric Posterior Fossa Tumor Surgery: A Comparative Analysis

**DOI:** 10.3390/jcm14134674

**Published:** 2025-07-02

**Authors:** Çağlar Türk, Umut Tan Sevgi, Sinan Bahadır, Mahmut Çamlar, Füsun Özer

**Affiliations:** 1 Department of Neurosurgery, University of Health Sciences, Izmir City Hospital, Izmir 35540, Turkey; usevgi@wisc.edu (U.T.S.); mahmut.camlar@sbu.edu.tr (M.Ç.); fusun.ozer@sbu.edu.tr (F.Ö.); 2 Department of Neurosurgery, Faculty of Medicine, Baskent University, Ankara 06790, Turkey; sinan.bahadir@baskent.edu.tr

**Keywords:** autograft, xenograft, duraplasty, posterior fossa, tumor

## Abstract

**Background/Objectives**: We aimed to review pediatric patients who underwent surgical treatment for posterior fossa tumors and to share our experience with the various types of dural grafts used in these patients. **Methods**: We carried out a retrospective study on pediatric patients who received surgical treatment for posterior fossa tumors and underwent duraplasty using either an autograft or a xenograft from January 2018 to December 2022. Data were gathered from patients’ medical records, encompassing demographic details. Additional information included tumor locations and the extent of resection. Factors such as postoperative complications like meningitis, pseudo-meningocele, and hydrocephalus were also noted. **Results**: Our cohort included 50 patients, 13 of whom underwent surgeries with autografts and 37 had xenografts. The patients’ tumors were in various areas, including intraventricular or those extending into the ventricle (31) and intracerebellar (17) and extra-axial (2) cases. Subtotal resection occurred in 8 cases, near-total resection in 9, and gross-total resection in 33. Postoperatively, meningitis occurred in 12 patients, pseudo-meningocele in 13, and hydrocephalus in 10, with 9 requiring V/P placement. **Conclusions**: In conclusion, techniques for dural closure hold great significance in neurosurgery, particularly during pediatric posterior fossa surgeries. Although the modest size of the autograft cohort limited statistical power, our epidural onlay fascia lata autograft produced fewer postoperative complications than the bovine xenograft and achieved outcomes comparable to those reported for watertight closure.

## 1. Introduction

About 70% of central nervous system tumors in children arise from the posterior fossa, and cerebrospinal fluid (CSF) leakage is among the most frequent complications following surgery for pediatric posterior fossa tumors [1]. This leakage can arise from disrupted natural CSF flow caused by tumor locations, the telovelar membrane opening in tumors in the fourth ventricle, poor dural closure, and the potential effects of gravity [2]. This can result in slower wound healing, infections, pseudo-meningocele development, extended hospital stays, and serious health risks such as meningitis [3]. Consequently, effectively closing the dura is crucial. Nevertheless, suturing the dura can be difficult because of its shrinkage over time, the effects of electrocautery, its relatively deep location, and potential dura defects [3].

To avoid CSF leakage after posterior fossa surgery, the literature suggests additional measures like preoperative and postoperative external ventricular drainage (EVD) or shunt placement, along with perioperative duraplasty techniques [4,5,6,7]. Duraplasty is crucial for preventing CSF leakage, as it establishes a reliable barrier between the intradural and extradural compartments [4]. Consequently, employing dural grafts has become increasingly essential to prevent CSF leaks [5]. Although many studies have explored this issue, a conclusive solution is still out of reach due to reported variations in CSF leakage rates across different research efforts, each utilizing unique methods [6,7,8,9].

In posterior fossa surgeries, duraplasty can utilize materials like autografts, allografts, or xenografts. The autograft is viewed as the gold standard [3,10]. While duraplasty using an autograft offers better outcomes regarding immune response, infection rates, and cost, it also presents drawbacks, including the requirement for extra incisions and longer surgery time [10,11]. Additionally, acquiring grafts from the pericranium is often challenging in posterior fossa surgeries [3]. Animal-derived xenografts are more widely available and cost-effective than allografts, which depend on limited donor availability. Moreover, xenografts, especially from the bovine pericardium, better mimic the mechanical properties of the native dura mater and have a lower risk of disease transmission than allografts due to their processing methods [12,13,14]. Choosing the correct type of durograft for duraplasty remains a contentious issue, with each method offering distinct advantages and disadvantages.

We conducted a retrospective review of pediatric patients who underwent surgery for posterior fossa tumors at our center, sharing our experiences with various dural-type grafts.

## 2. Materials and Methods

### 2.1. Study Design

We carried out a retrospective study on pediatric patients who received surgical treatment for posterior fossa tumors and underwent duraplasty using either an autograft or a xenograft from January 2018 to December 2022. The institutional ethics committee sanctioned this research (Number: 2022/04-12, Date: 15 April 2022), and informed consent was obtained from the patients’ legal guardians. Excluded from our study were patients over 18 years old, those with a history of posterior fossa surgery, patients with insufficient medical records, and those with allograft material used in their surgery. Initially, commercially available xenografts (DuraGen^®^) were preferred, but due to difficulties in obtaining xenografts and limited commercial access during the study period, autografts (fascia lata) were used in subsequent cases. All surgical procedures were performed by the same pediatric neurosurgery team. The number of surgical team members reported only includes attending surgeons and surgical residents directly involved in performing the operation, excluding nurses, technicians, and ancillary staff. We excluded patients who underwent watertight closure.

### 2.2. Data Collection

Data were gathered from patients’ medical records, encompassing demographic details. Additional information included the duration of preoperative hospital stays, tumor locations (intraventricular, intraparenchymal, or extra-axial), the number of surgical team members, and the extent of resection, categorized as subtotal (51–90%), near total (91–99%), or gross total (>99%). Factors such as blood loss, surgery duration, the placement of intraoperative EVD, and postoperative complications like meningitis, pseudo-meningocele, CSF leak, and hydrocephalus were also noted. Additionally, data regarding patients’ dependence on CSF diversion, occurrences of intraoperative air embolism, and nonsurgical complications were recorded. The necessity for ventriculoperitoneal (VP) shunts was also assessed, determined by persistent symptomatic hydrocephalus, worsening ventricular enlargement from postoperative imaging, or clinical deterioration requiring permanent CSF diversion. Intraventricular tumors were defined as those with openings into the ventricle. Postoperative infections were identified through wound infections (either superficial or deep), meningitis, or ventriculitis within the postoperative period. Meningitis was determined based on the detection of pathogens in CSF culture or direct examination, accompanied by at least one of the following symptoms: fever, meningeal irritation, neck stiffness, or general irritability. The development of pseudo-meningocele was evaluated through radiological imaging or observed during a subsequent surgery.

### 2.3. Surgical Technique

#### Autograft Preparation and the Duraplasty Technique

Posterior fossa surgeries are conducted with patients in prone or ¾ prone oblique positions. After sterile preparations, the skin is incised, and the fascia lata is accessed through a blunt dissection of the subcutaneous fat. The fascia lata is carefully separated from adjacent tissues, and a graft measuring at least 3 × 3 cm is obtained. This graft is stored in a 0.9% NaCl solution until the duraplasty procedure.

**Figure 1:**
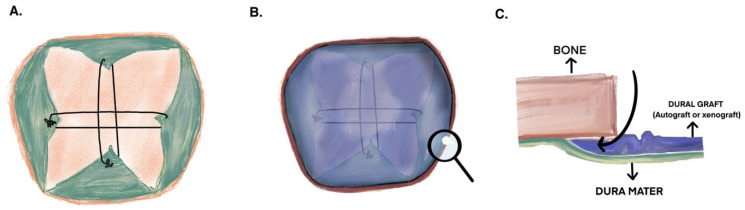
(**A**) An illustration showing bridging sutures threaded through the closest points of the dura, which is opened in a plus shape. These sutures create tension to minimize the area and prevent the dura graft from sagging. (**B**) The dura graft, cut to match the craniotomy dimensions, is depicted as an onlay. A magnifying glass on the right highlights the area focused on in the next figure. (**C**) When observing horizontally at the bone, the insertion of the dura graft into the epidural space is visible. The dissector prepares the epidural space for the dura graft.

The duraplasty was conducted utilizing an onlay technique. First, we brought together the shrunken dura sheets with crosswise sutures at their closest points. These sutures help secure the graft and generate tension between the dura sheets, thereby reducing the gap that requires closure (Figure 1A). Following this, we trimmed and reshaped the harvested graft or autograft to match the craniectomy size and positioned it on the suture-supported base we created (Figure 1B). Subsequently, we placed it as an onlay in the epidural space using a microdissector, carefully ensuring it did not induce any epidural bleeding (Figure 1C). According to our institution’s standard procedure, if we identify a CSF leak or it occurs after a Valsalva maneuver, we apply wet Gelfoam to that site for pressure on the graft. We refrain from using any adhesive agents to avoid fibroblast formation. If the graft appeared highly unstable, one or two fixation sutures were placed to prevent displacement; however, the primary fixation was achieved by tucking the graft into the epidural space. Pericranium grafts were not employed, as our institutional practice favors fascia lata due to its ease of harvesting, greater thickness, resistance to fragmentation, and ability to be more securely tucked into the epidural space. Additionally, the fascia lata provides the reliably larger and more suitable dimensions necessary for stable fixation using a circumferential epidural overlay technique.

### 2.4. Statistical Analysis

Statistical analysis was conducted using IBM SPSS (Statistical Package for the Social Sciences) Statistics Version 24.0. Categorical variables are represented as frequencies, while continuous variables are presented as means and standard deviations. The chi-square test was employed to compare categorical variables, and the Mann–Whitney U test was used for continuous variables. A significance level of *p* < 0.05 was deemed statistically significant.

## 3. Results

### 3.1. Patients’ Characteristics

Our cohort included 50 patients, 13 of whom underwent surgeries with autografts and 37 had xenografts. We utilized DuraGen^®^ Plus Adhesion Barrier Matrix (Integra LifeSciences, Princeton, NJ, USA), a xenograft derived from the bovine deep flexor (Achilles) tendon, as the dural substitute. The study population had a mean age of 7.46 ± 5.04 years, with 20 females (40%).

The patients’ tumors were in various areas, including intraventricular or extending into the ventricle (*n* = 31; 62%), intracerebellar (*n* = 17; 34%), and extra-axial (*n* = 2; 4%). Patient baseline characteristics are summarized in Table 1.

### 3.2. Between-Group Comparisons

Both study groups, the autograft and xenograft, exhibited similar age and gender distributions. While the mean GCS scores were comparable across both groups, a higher proportion of patients in the xenograft group experienced mild neurological impairment (*p* = 0.048). The tumor locations did not vary between the two groups. The average number of surgeons involved was 3.5 ± 0.8 in the autograft group and 3.7 ± 0.8 in the xenograft group. The rates for gross total, near total, and subtotal excisions were similar, with gross-total resection achieved in over 60% of cases in both categories. The average surgery duration was 245.4 ± 55.4 min for the autograft group and 244.2 ± 58.1 min for the xenograft group (*p* = 0.921). Although one case in the autograft group required EVD placement compared to seven in the xenograft group, this difference was not statistically significant (*p* = 0.342). The postoperative intensive care unit stay averaged 2.8 ± 2.4 days for the autograft group and 8.4 ± 17.0 days for the xenograft group, with no statistically significant difference (*p* = 0.158).

Most tumors in the autograft group were pilocytic astrocytomas (53.8%), whereas medulloblastoma was slightly less frequent than astrocytoma in the xenograft group (29.7% vs. 32.4%). Regardless, the distribution of pathological diagnoses was statistically similar in both groups (*p* = 0.637).

In the xenograft group, there was a single instance of air embolism during surgery. The rate of postoperative meningitis was 29.7% in this group, compared to 7.7% in the autograft group; however, this difference was not statistically significant (*p* = 0.110). No postoperative CSF fistulas occurred in the autograft group, while 21.6% (*n* = 8) were noted in the xenograft group, again lacking statistical significance (*p* = 0.067). Likewise, pseudo-meningocele was more frequently observed in the xenograft group than in the autograft group (32.4% vs. 7.7%), but this difference was also insignificant (*p* = 0.080). In the autograft group, one patient developed postoperative hydrocephalus and needed a VP shunt. Conversely, 24.3% (*n* = 9) of patients in the xenograft group experienced hydrocephalus, with 21.6% (*n* = 8) requiring shunt placement, showing no statistically significant difference. Nonsurgical complications were found in 23.1% (*n* = 3) of the autograft patients and 13.5% (*n* = 5) of the xenograft patients (Table 2).

## 4. Discussion

In our series, the fascia lata autograft applied as an onlay with bridging sutures showed lower rates of CSF leakage, meningitis, pseudo-meningocele, and hydrocephalus and a shorter ICU stay than the bovine xenograft. Because the autograft group was small, these differences did not reach statistical significance. Still, the favorable trend and the straightforward nature of this technique are consistent with earlier reports and should be re-examined in larger, balanced studies.

The dura mater is a vital protective membrane that shields brain tissue, making its integrity critical. Defects in this layer can result from trauma, tumor infiltration, surgical interventions, or other medical procedures, possibly causing complications like seizures, meningitis, CSF leaks, cerebral herniation, and pseudo-meningocele. Although small defects can often be repaired with sutures, major damage typically requires the augmentation or replacement of the damaged dura mater [4]. Neurooncological surgeries, particularly in the posterior fossa, often require grafts. During these procedures, the prolonged exposure of the dura to heat from the surgical microscope’s light, combined with extensive electrocautery for managing large occipital sinuses, can result in the dura mater shrinking and becoming dry and thin. This makes primary suturing for dural closure extremely difficult, so grafting is necessary [7].

Choosing the appropriate dural graft has been debated for years. Options include traditional grafts, such as autologous, allogeneic, and xenogenic grafts, and polymeric ones, such as synthetic and natural grafts [15].

Natural polymers provide excellent biocompatibility and encourage connective tissue development; however, their quality can vary between batches, degrade quickly, and have limited mechanical strength. In contrast, synthetic materials present a stable, inert option that can be produced continuously with uniform characteristics. Still, they fail to integrate with the native dura through vascular and epithelial fusion like a biological graft [12].

Neurosurgeons frequently utilize autologous materials such as the fascia lata, pericranium, galea pericranium, temporalis fascia, nuchal ligament, and fat packings as substitutes for the dura mater. These options are preferred due to their non-toxic and non-immunogenic properties, availability, and cost-effectiveness. Nonetheless, harvesting pericranium during posterior fossa surgeries poses significant difficulties. Additionally, its delicate and thin structure complicates surgical handling and suturing [12]. The nuchal ligament works well for suboccipital median approaches, but it is not suitable for other techniques such as paramedian, retro sigmoid, and far lateral approaches. Conversely, harvesting fascia lata necessitates an additional incision, which introduces another potential site for surgical complications.

Xenografts originate from porcine, equine, bovine, or other animal tissues. These biological tissues eliminate the need for harvesting from the patient while ensuring secure dural closure. Although there is a minimal risk of infection transmission, it is extremely low. Additionally, some xenografts often do not need suturing, which helps decrease operative time and aids in positioning the graft in challenging areas [12]. Danish and colleagues noted in the literature that using non-sutured xenografts resulted in shorter operating room times than allografts. This approach also helps reduce anesthesia-related complications, medical risks, and costs [16]. These grafts can also be utilized in more complex surgical sites and serve as smaller components.

Engineered collagen grafts, such as DuraGen^®^ (Integra), can be used suturelessly [17,18,19]. Conversely, non-autologous grafts may lead to specific complications such as aseptic meningitis and bacterial infections [20]. Many studies have shown that watertight dural closure with autologous grafts is superior to xenografts [3,21]. In our study, even within the cohort that received autografts using a non-watertight overlay technique, the recorded meningitis rate was 7.7%. This figure is an improvement compared to the xenograft group, which exhibited a meningitis risk of 29.7%. Although this reduction in meningitis risk (7.7% vs. 29.7%) did not reach statistical significance—likely reflecting the limited power of the autograft cohort (*n* = 13)—the magnitude of this difference remains clinically relevant and warrants confirmation in larger studies. Additionally, a retrospective analysis by Zaben et al., encompassing 68 pediatric patients with posterior fossa tumors, found that the incidence of surgical site infections was approximately 22% after watertight dural closure using DuraGen^®^ (Integra) and Tisseel fibrin sealant (Baxter) [22]. Research indicates that these rates are notably reduced in groups utilizing autografts in the posterior fossa surgeries [21].

We chose fascia lata for autografts because it can produce a larger dural graft when necessary. If we are not aiming for a watertight closure, we do not harvest pericranium. For a single-layer onlay graft placed without sutures, the patch must remain intact and be wide enough to tuck beneath every edge of the craniotomy. Also, we believe that the pericranium is more difficult to peel off in pediatric cases, especially in infants. Hence, this option enables two surgeons to operate simultaneously at separate incisions, which does not extend the duration of the surgery and is not technically challenging. During duraplasty, the dura can be sutured primarily to form a watertight seal, with dural grafts applied either as an onlay, inlay, or a combination of both approaches. Furthermore, adhesives such as TISSEL can be effectively utilized [14,15]. The non-watertight closure of the dura presents specific advantages [18,23]. The first is a decrease in the operation time. Studies suggest that non-watertight closures save time compared to watertight closures [24]. Second, evidence indicates that suture materials can provoke a tissue reaction, alongside the potential for complications like bleeding or parenchymal injury during dural suturing. A study by Dafford et al. also revealed that CSF leakage may occur through the needle insertion site passes [25]. We layered the dura and inspected for any visible leakage from the bosom. During our study, we harvested our autografts from the fascia lata while the case was ongoing and applied our grafts onlay. The operation times in both groups were comparable since the grafts were used as an overlay. The critical factor was that the graft size should be no smaller than the craniectomy size.

Pseudo-meningocele development is among the most frequent postoperative complications in posterior fossa surgery. These pseudo-meningoceles can be associated with symptoms or present radiologically [26]. While rates differ across various case studies, the consensus suggests that pseudo-meningocele formation is less frequent in cases utilizing autografts [21]. In a 174 pediatric posterior fossa tumors study, Steinbok et al. reported a 33% rate of clinical or radiological postoperative pseudo-meningocele formation. They found that applying tissue adhesive, dural grafts, and EVD did not influence this rate [7]. In a study with 214 pediatric patients who had posterior fossa tumors, Gecici et al. found that using postoperative EVD increased the risk of developing pseudo-meningocele and CSF leakage after achieving watertight closure with autografts [3]. Although pseudo-meningocele occurred in only 7.7% of the autograft cohort versus 32% of the xenograft cohort—an absolute risk reduction of 24.7% (95% CI −45.6% to −3.8%)—this comparison did not achieve statistical significance, most likely because the autograft group was small (*n* = 13); nonetheless, the magnitude of the observed difference is clinically noteworthy and should be explored in larger series. Likewise, no postoperative CSF fistula occurred in the autograft group (0/13; 95% CI 0–20.6%), whereas the xenograft group showed a 21.6% rate (8/37; 95% CI 11.4–37.2%), giving an absolute risk reduction of 21.6 percentage points (95% CI −34.9% to −8.3%). Although this failed to reach statistical significance (*p* = 0.067), the complete absence of fistula with autografts signals a potential clinical advantage that merits validation in larger cohorts. Research indicates that following posterior fossa surgery, pseudo-meningocele rates vary from 4% to 28%, whereas CSF leakage rates range from 2% to 23%. Both complications occur more often in children [6,8,9,27]. In our study, the pseudo-meningocele rate in the xenograft group was higher than in the other literature series because we classified radiological and clinical manifestations as pseudo-meningocele. Additionally, we considered any visible pseudo-meningocele formation in the MRI scans at 3 months, 6 months, or 1 year as an instance of pseudo-meningocele. Moreover, since prior reports suggest that craniectomy is more likely to lead to pseudo-meningocele formation compared to craniotomy, the choice to perform craniectomy on our patients might have further contributed to this elevated rate [28].

Following surgery in the posterior fossa, hydrocephalus and the need for shunts are more common, particularly when the tumor is situated in the fourth ventricle or causes significant compression of the ventricular system [3]. Although previous studies have shown that between 10% and 30% of patients who underwent posterior fossa surgery eventually require a permanent shunt [10,21]. The currently available literature does not appear to show any significant relationship between the type of duraplasty graft and postoperative hydrocephalus or shunt dependency [7]. In our cohort, the limited size of the autograft group may have prevented us from detecting a statistically significant difference between graft materials. However, we observed a trend indicating that autologous grafts might better preserve CSF circulation. This potential advantage could stem from variations in tissue compatibility or the inflammatory responses linked to different graft types, which may influence CSF flow dynamics. Additional research with larger sample sizes will be essential to confirm this finding. Nonetheless, we believe the primary factors influencing the development of hydrocephalus and the consequent need for shunting are the extent of preoperative obstruction and the tumor’s relation to the fourth ventricle [10]. In addition, preoperative EVD/endoscopic intervention strategies and the surgical excision of the tumor as much as possible may play a key role in reducing the patient’s postoperative hydrocephalus and shunt dependency [3].

Postoperative meningitis in children is an important and potentially life-threatening complication after posterior fossa tumor surgery, caused by direct surgical contamination, a foreign body, or a postoperative CSF leak [21,29]. Postoperative meningitis rates in the literature vary considerably [21]. In their study involving 66 pediatric patients who underwent tumor resection using a suboccipital midline approach, Wang et al. separated the patients into two groups: autografts and xenografts. They found that the rate of meningitis was 2.6% among the autograft patients, compared to 11% in the xenograft group, which was deemed statistically significant [10]. Zhao et al. investigated 123 patients with posterior fossa tumor surgery, dividing them into autograft and xenograft groups. They found that the rate of meningitis was 8.33% in the autograft group, compared to 24% in the xenograft group. Notably, every culture-positive case in their research occurred in the artificial dura group [21]. In our series, meningitis occurred in 7.7% of the autograft group and 29.7% of the xenograft group. Nevertheless, the difference in infection rates between these groups was not statistically significant. While opinions vary in the literature on the complication rates of autologous versus non-autologous grafts, it is widely accepted that autografts offer more advantages in this context [8,12].

While ICU stay was shorter in the autograft group by roughly 5.6 days, the small sample size in that group and the large variance observed in the xenograft group limited the statistical power of this secondary analysis. Consequently, the non-significant *p*-value should not be interpreted as evidence of clinical equivalence; it may simply reflect insufficient power to detect a true difference. In the literature, studies focus predominantly on infectious and CSF-related complications [2,3]. However, our clinical observations suggest that the incidence of secondary events such as meningitis and VP shunts in patients with xenografts may prolong intensive care periods. Future studies with larger, more balanced cohorts will be valuable for clarifying whether graft choice truly influences postoperative ICU stay.

The main limitation of this study is the modest size of the autograft subgroup (*n* = 13). This reduces statistical power and may explain why significant absolute risk reductions did not reach traditional significance thresholds. As a retrospective, single-center study, our dataset cannot capture extremely late or subclinical events, and long-term functional or cost outcomes are outside the scope of this analysis. In addition, tumor localization, which may theoretically influence CSF-leak rates, was not statistically analyzed between groups. Nevertheless, routine cerebrospinal fluid drainage from the cisterna magna was performed in all cases, combined with the selective use of preoperative external ventricular drainage based on clinical indication, likely minimized the impact of tumor location on CSF leakage.

In conclusion, techniques for dural closure hold great significance in neurosurgery, particularly during pediatric posterior fossa surgeries. Although the autograft cohort showed numerically fewer complications, this difference did not reach statistical significance—likely because the autograft sample was small. Our data indicate that an epidurally seated onlay fascia lata achieves postoperative complication rates comparable to those reported for watertight dural closure. By eliminating the suturing step, the technique shortens dural-closure time and thus offers a practical alternative to xenograft duraplasty. More extensive studies involving larger patient populations are necessary to explore this further.

## 5. Conclusions

Watertight dural closure with fascia lata autografts remains the gold standard in pediatric posterior fossa surgery. Nonetheless, techniques for dural repair continue to play a critical role. In our series, the autograft cohort showed numerically fewer complications, but this difference did not reach statistical significance—most likely because the autograft sample was small. Our data indicate that an epidurally seated onlay fascia lata graft achieves postoperative complication rates comparable to those reported for watertight closure. Because it eliminates suturing, the method shortens dural-closure time and offers a practical alternative to xenograft duraplasty. Larger multicentre studies are required to verify these observations.

## Figures and Tables

**Table 1:** Baseline characteristics of pediatric patients undergoing posterior fossa surgery with autograft or xenograft duraplasty.

		Autograft (*n* = 13)	Xenograft (*n* = 37)
Age (year)		7.6 ± 3.6	7.4 ± 5.5
Gender (M:F)		8:5	22:15
Tumor location			
	Intraventricular	8 (61.5)	23 (62.2)
	Intraparenchymal	5 (38.5)	12 (32.4)
	Extra-axial	0 (0.0)	2 (5.4)
Amount of resection			
	Subtotal	2 (15.4)	6 (16.2)
	Near total	3 (23.1)	6 (16.2)
	Gross total	8 (61.5)	25 (67.6)
Perioperative EVD		1 (7.7)	7 (18.9)
Pathology			
	Medulloblastoma	2 (15.4)	12 (32.4)
	Pilocytic astrocytoma	7 (53.8)	11 (29.7)
	Ependymoma	2 (15.4)	7 (18.9)
	Epidermoid	0 (0.0)	2 (5.4)
	Embryonal tumor (WHO IV)	1 (7.7)	2 (5.4)
	Diffuse astrocytoma (IDH mutant) (WHO III)	1 (7.7)	1 (2.7)
	Low-grade glial tumor	0 (0.0)	1 (2.7)

M: male, F: female, EVD: external ventricular drain, WHO: World Health Organization, and IDH: isocitrate dehydrogenase.

**Table 2:** Comparison of patients using autografts and xenografts for duraplasty.

		Autograft (*n* = 13)	Xenograft (*n* = 37)	*p*
Duration of surgery (min.)		245.4 ± 55.4	244.2 ± 58.1	0.921
Postoperative ICU admission (day)		2.8 ± 2.4	8.4 ± 17.0	0.158
Postoperative meningitis		1 (7.7)	11 (29.7)	0.110
Postoperative CSF fistula		0 (0.0)	8 (21.6)	0.067
Postoperative pseudo-meningocele		1 (7.7)	12 (32.4)	0.080
Postoperative hydrocephalus		1 (7.7)	9 (24.3)	0.197
V/P shunt		1 (7.7)	8 (21.6)	0.261
Additional surgical complications				0.549
	Air embolism	0 (0.0)	1 (2.7)	
Nonsurgical complications				0.238
	Hyponatremia	1 (7.7)	0 (0.0)	
	Urinary tract infection	1 (7.7)	0 (0.0)	
	Keratitis	1 (7.7)	0 (0.0)	
	Blepharitis	0 (0.0)	1 (2.7)	
	Catheter infection	0 (0.0)	1 (2.7)	
	Seizure	0 (0.0)	1 (2.7)	
	Perioperative acidosis	0 (0.0)	1 (2.7)	
	Septicemia	0 (0.0)	1 (2.7)	

Continuous data are given as mean ± standard deviation, and categorical data are given as frequency (percentage).

## Data Availability

The data presented in this study are not publicly available due to privacy and ethical restrictions. However, they can be made available from the corresponding author upon reasonable request and with permission from the respective ethics committee.

## Abbreviations

The following abbreviations are used in this manuscript:

CSF	Cerebrospinal Fluid
GCS	Glascow Coma Score
EVD	External Ventricular Drain
ICU	Intensive Care Unit
VP	Ventriculoperitoneal
